# The Non-Linear Elasticity of Unidirectional Continuous Carbon Fibre-Reinforced Composites and of Carbon Fibres

**DOI:** 10.3390/ma17010034

**Published:** 2023-12-21

**Authors:** Vincent Keryvin, Adrien Marchandise

**Affiliations:** 1 UMR CNRS 6027, IRDL, Université Bretagne Sud, F-56321 Lorient, France; 2 Avel Robotics, F-56100 Lorient, France; marchandise@avelrobotics.com

**Keywords:** polymer composites, CFRP, carbon fibres, non-linear elasticity, bending tests, stiffening, softening, mechanical properties, AFP

## Abstract

A database of non-linear elastic parameters in axial tension and compression is provided for continuous carbon fibre polymer composites and carbon fibres of different stiffnesses. Composite laminates manufactured by conventional or automated processes are tested in bending, and parameters are extracted for strains of less than 0.5%. While fibre composites with fibres of standard and intermediate moduli exhibit a stiffening of ∼15 GPa/% (of strain) and a softening of ∼20 GPa/%, those with high-modulus carbon fibres exhibit much higher values of ∼50 GPa/% for both. This database is useful for designing composite structures in a stiffness-based design and for correlating the processing of carbon fibres with their nanostructure and induced properties. The latter is discussed in terms of reorientation of crystallites of graphene sheets vis-à-vis the carbon fibre axis during loading.

## 1. Introduction

Today, more and more mechanical structures are made from fibre composites, mainly because of their very high specific mechanical properties, such as stiffness, strength and fatigue endurance [1]. Continuous carbon fibre polymer composites (CFRP) are known to exhibit limited compressive strength relative to tensile strength in the fibre direction, due to initial fibre waviness created mainly during curing, which prematurely triggers fibre buckling, leading to catastrophic failure [2,3]. These materials also exhibit some non-linear elasticity in tension and compression in the fibre direction [4,5]. Under tensile loading, they stiffen, while under compression, they soften.

Carbon fibres are highly anisotropic materials. The longitudinal stiffness of most PAN-based (Polyacrilonitrile precursor) fibres is of the order of [200–600] GPa, while their transverse stiffness is well below 20 GPa [6,7]. The non-linear elastic behaviour of carbon fibres themselves was reported as early as the 1960s [8,9]. This behaviour is linked to the reorientation of the graphene crystallites in the direction of the tensile load [10]. During compression, the carbon fibres break due to the buckling of these crystallites [11,12].

At the scale of the unidirectional (UD) ply, the possible origins of the elastic non-linearity are the carbon fibres themselves, their waviness or the behaviour of the matrix. The latter is excluded because the stiffness contrast between fibres and polymer matrices is at least 50. Keryvin et al. [13] have shown that the contribution of fibre waviness is almost zero for strains of less than 0.5%. The elastic non-linearity of UD plies is therefore entirely dictated by that of the carbon fibres.

The practical consequences of the elastic non-linearity of UD plies include the overestimation of compressive strength in bending tests [14] or the overestimation of structural stiffness, for example, in the stiffness-based design of masts for racing yachts [15] or sandwich structures. Fundamental implications include the influence of carbon fibre processing on their nanostructure (voids, orientation and distribution of graphene layers, heterogeneity…), and hence on their properties [16,17,18].

Characterising this elastic non-linearity requires either tests on carbon fibres with tensile tests on individual fibres [19] or on tows [9], and compressive tests that are extremely difficult to perform, or tests on UD plies in longitudinal tension and compression [5]. Flexural tests were also used to complement tension tests and identify the non-linear behaviour in compression [20,21,22]. Keryvin et al. [14] proposed an experimental method based on a single bending test to rapidly extract the non-linear elastic characteristics of UD plies. They proved to be consistent with the tests mentioned above, provided that the range of strains considered was the same [13].

The aim of this work is twofold. Firstly, we present elastic non-linearity values for a large CFRP database including SM (standard-modulus), IM (intermediate-modulus) and HM (high-modulus) carbon fibres, manufactured manually or by Automated Fibre Placement (AFP). This database can be used to design laminated composite structures in terms of stiffness. On the other hand, we use this database to extract the elastic non-linearity of the carbon fibres themselves, in tension and compression, which can be used in processing–structure–property relationships of carbon fibres. The results are discussed at the light of the ultra-microstructural characteristics of carbon fibres.

## 2. Materials and Methods

### 2.1. Materials and Experiments

Composite plates are laminates with 32 unidirectional plies (UD). Each UD ply is composed of an epoxy resin matrix and carbon fibres (standard modulus—SM, intermediate modulus—IM and high modulus—HM). The prepreg batch had a resin weight content of ∼35% and fibre weight of mostly ∼300 g/m${}^{2}$ on average for all composites. The ply thickness is mostly ∼300 µm. The fibre volume fraction is ∼55%. The laminates are composed of 11 blocks of plies with the following stacking sequence: [+45°|0${}_{6}$°|+45°/−45°|0${}_{4}$°|−45°|0${}_{2}$°]${}_{S}$. This lay-up is rather classical for racing yachts [23]. Some plates were manufactured classically, and some other plates were fabricated by AFP with a C1-Coriolis Composites AFP robot (Queven, France). The plates were manufactured by AVEL Robotics (The influence of the manufacturing process was studied by Marchandise et al. [24]. While AFP plates exhibited higher performances in strength, the elastic behaviour was not affected).

The stacking sequence used for the materials, from the tool surface to the bagging materials, was as follows: a 12 mm thick aluminium alloy tool, a non-porous release film, the pre-impregnated carbon/epoxy stack, another non-porous release film, a 3 mm thick aluminium alloy caul plate, a polyester breather and a vacuum sealant. An intermediate vacuum debulking for 20 min at less than 1 bar was applied every 3 plies, except when using AFP, where the process was carried out in a single operation and the sole compaction force of the robot head was used to create the adhesion between the plies, without intermediate debulking.

The curing cycle consisted of a single thermal cycle in an autoclave. The prepreg stack was monitored and did not undergo any exothermic reactions. The maximum pressure and temperature were 7 bar and 120 °C, respectively (135 °C for the M81 resin), throughout the cycle. The plates were not deformed during demoulding and retained their flatness.

The plates were precision-machined by water-jet cutting into 500 × 30 × 10 mm${}^{3}$ samples. The thickness of each sample was measured using a caliper. The thickness of each block of plies was measured by optical microscopy (Olympus BX35, Tokyo, Japan). The samples proved to be perfectly symmetrical for this measurement accuracy. (It was found that non-symmetrical plates could not be used to characterise the non-linear elastic behaviour, unless a more sophisticated methodology was employed) [24].

**Figure 1:**
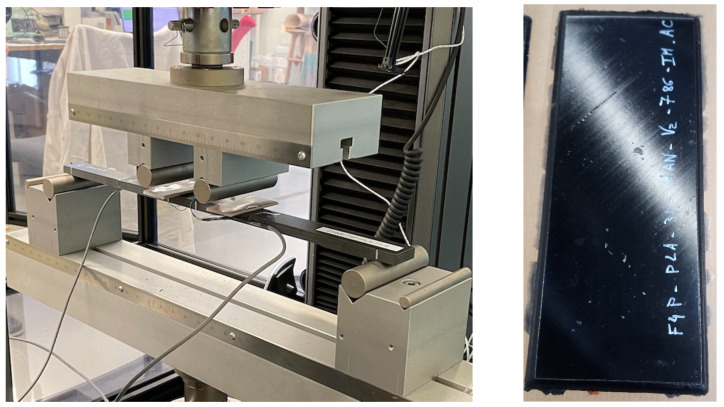
(**Left**) Four -point bending test apparatus on CFRP laminates. (**Right**) CFRP plate after curing in an autoclave.

Four-point bending tests were carried out with a universal testing machine (Instron 5567, 30 kN load cell, Norwood, MA, USA) using a distance of 90 mm between the upper rollers (25 mm diameter), where pure bending occurs, and a distance of 460 mm between the lower rollers. Polyethylene plates were placed under the rollers to minimise stress concentrations. Single-axis strain gauges (10 mm long—Kyowa, Japan) were bonded to both the compression and tension sides. The sudden failure occurred between the top rollers. Further details are available in Mechin et al. [25] and a picture of the testing apparatus, as well as CFRP plate after curing, is shown in Figure 1.

At least three samples were tested with the mould (tool) side in compression, and three with the other (vacuum) side in compression. The force on the assembly and the two strain gauge signals ($\epsilon_{c}^{g}$ in compression and $\epsilon_{t}^{g}$ in tension) were recorded during loading and synchronised. The position of the neutral axis, λ, for which the axial strain (beam direction) was null, was calculated following Euler–Bernoulli kinematics as well as the strain gradient γ by Equation (1):

(1) $$\lambda =-\frac{h}{2}\frac{\epsilon_{c}^{g}+\epsilon_{t}^{g}}{\epsilon_{c}^{g}-\epsilon_{t}^{g}}\;;\;\gamma =\frac{\epsilon_{c}^{g}-\epsilon_{t}^{g}}{h},$$

### 2.2. Data Analyses

The aim of this section is to explain how, in a single bending test on CFRP laminates, linear and non-linear elastic parameters are identified. The longitudinal elastic modulus of the ply, $E_{\mathrm{UD}}$, depends on the type of ply in the laminate. For the 0° plies (beam direction), it is assumed to depend linearly [5,20,22,26] on the longitudinal strain $\epsilon_{\mathrm{UD}}$, as given by Equation (2):

(2) $$\begin{array}{cc} \mathrm{Compression}\;(C):\; & E_{\mathrm{UD}}^{C}\left(\epsilon_{\mathrm{UD}}\right)=E_{\mathrm{UD}}^{C0}+\alpha_{\mathrm{UD}}\times \epsilon_{\mathrm{UD}},\\ \mathrm{Tension}\;(T):\; & E_{\mathrm{UD}}^{T}\left(\epsilon_{\mathrm{UD}}\right)=E_{\mathrm{UD}}^{T0}+\beta_{\mathrm{UD}}\times \epsilon_{\mathrm{UD}}, \end{array}$$

where $E_{\mathrm{UD}}^{T0}$ and $E_{\mathrm{UD}}^{C0}$ are the initial tensile and compressive moduli and $\beta_{\mathrm{UD}}$ and $\alpha_{\mathrm{UD}}$ are the coefficients of linear dependence of tensile and compressive moduli with strain. Let *M* denote the bending moment and *N* the normal force at the middle of a specimen undergoing four-points bending. They are computed using the classical laminate theory with a Euler–Bernoulli kinematics assumption, and they depend on these four parameters introduced. Their values are found by minimizing the error between *M* and the applied bending moment throughout the test as well as the constraint of a null normal force *N* [14,20,21,22].

The methodology for extracting the elastic properties of the UD plies in the beam direction is described by Keryvin et al. [14]. Readers are invited to refer to it for further details (let us notice that this methodology was validated by finite element analyses [14]). It consists of minimising the relative error between the experimental bending moment and an estimated value of this moment. This procedure involves four parameters: the initial moduli in tension ($E_{\mathrm{UD}}^{T0}$) and in compression ($E_{\mathrm{UD}}^{C0}$), as well as the linear decrease in the elastic modulus in tension ($\beta_{\mathrm{UD}}$) and in compression ($\alpha_{\mathrm{UD}}$), with strain.

To be able to compare the different types of carbon fibres, which exhibit different tensile failure strains, the range of deformation is chosen to be [0.1–0.5]% for all tests since CFRPs with HM fibres fail at around 0.5% during bending tests [25].

## 3. Results

**Figure 2:**
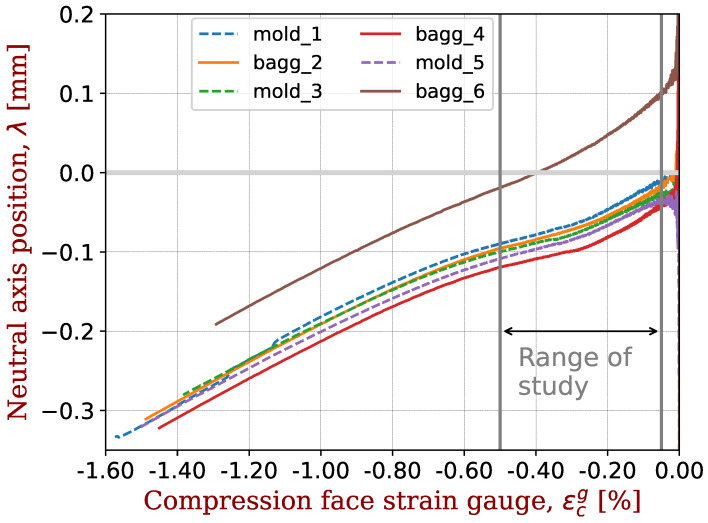
Displacement (shift) of the neutral axis during the bending test towards the tensile side of the specimen as a function of the strain gauge signal on the compression side. The carbon fibre is IM2C and the epoxy resin is M81. Three samples are tested with the mould side in compression (labelled “mold”) and three with the vacuum bagging side in compression (labelled “bagg”). As an example of data filtering, sample #6 is removed from the data analysis. The strain range used for this study is also indicated.

The shift of the neutral axis during the loading is illustrated in Figure 2. Originally positioned at the mid-axis, the neutral axis gradually shifts towards the tensile side of the specimen during bending. Keryvin et al. [13] showed that for a composite with glass fibres, there is no such shift during bending: the shift comes from the carbon fibre. This offset is therefore considered to be an indicator of the elastic non-linearity of the carbon fibres.

Table 1 shows the different properties computed by the methodology from Section 2.2 for different CFRPs: $E_{\mathrm{UD}}^{T0}$, $E_{\mathrm{UD}}^{C0}$, $\beta_{\mathrm{UD}}$ and $\alpha_{\mathrm{UD}}$. Matrices from suppliers include Se84LV and Se84nano2 (Gurit, Newport, UK), R374-1 (Structil, Vert-le-Petit, France), BT080, DT120 and DT124 (Delta-Preg, Sant’egidio Alla Vibrata, Italy), M79 and M81 (Hexcel, Les Avenières Veyrins-Thuellin, France), RV101 (Vitech, Châtillon, France), MR074 (Toray, Lacq, France). Carbon fibres include UTS50, IMS65 (Teijin, Osaka, Japan), IM2C (Hexcel, Les Avenières Veyrins-Thuellin, France), HR40 (Mitsubishi, Chichibu, Japan), T800G, T800S (Toray, Tokyo, Japan). Some fibres are present more than once to check a possible influence of the matrix. For example, fibre IM2C is used six times, and the results are extremely similar, indicating that there is no noticeable influence of the matrix on the elastic non-linearity of UD plies. The first observation from Table 1 is that the initial elastic moduli in compression $E_{\mathrm{UD}}^{C0}$ and in tension $E_{\mathrm{UD}}^{T0}$ are equal, unlike what is sometimes reported in the literature [27,28]. A certain degree of variation may sometimes be observed, mainly due to a limited number of samples or valid samples.

**Figure 3:**
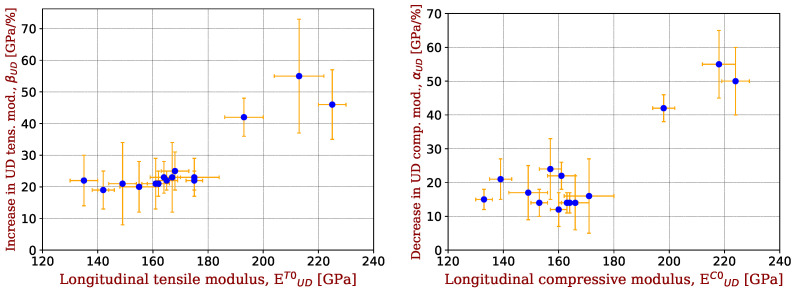
Non-linear elastic parameters of UD plies ($\beta_{\mathrm{UD}}$) in tension (**left**) and ($\alpha_{\mathrm{UD}}$) in compression (**right**), as a function of the initial tensile/compressive elastic modulus of UD ply for the different CFRP. Data are taken from Table 1.

Figure 3 shows the evolution of non-linear elastic parameters of UD plies ($\beta_{\mathrm{UD}}$ and $\alpha_{\mathrm{UD}}$) as a function of the initial elastic modulus of UD ply. For SM and IM fibres (E${}_{f}$ < 300 GPa), consistent $\beta_{\mathrm{UD}}$ values are ∼20 GPa/%, while they are ∼50 GPa/% for HM fibres, i.e., ∼2.5 times higher. For $\alpha_{\mathrm{UD}}$, there is more scatter with values between 12 and 22 GPa/% for SM and IM fibres vs. ∼50 GPa/% for HM fibres, i.e., ∼3 times higher. As a first approximation, carbon fibres have the same non-linear parameters in tension and compression. Those of HM fibres are far higher than those of IM fibres.

## 4. Discussion

### 4.1. Non-Linear Elastic Parameters of Carbon Fibres

Since the elastic non-linearity comes from the carbon fibre itself, not from the matrix nor the fibre waviness [14], we define two new parameters that are intrinsic to the fibres. They are computed according to Equation (3) in the range [0.1–0.5]% of strain from the parameters of UD plies in Table 1.

(3) $$\begin{array}{cccc} \mathrm{Tension}\;(T): & \sigma_{\mathrm{UD}}^{T} & = & E_{\mathrm{UD}}^{T0}\epsilon_{\mathrm{UD}}^{T}\left(1+\beta_{f}^{\prime }\times \epsilon_{\mathrm{UD}}^{T}\right),\\ \mathrm{Compression}\;(C): & \sigma_{\mathrm{UD}}^{C} & = & E_{\mathrm{UD}}^{C0}\epsilon_{\mathrm{UD}}^{C}\left(1+\alpha_{f}^{\prime }\times \epsilon_{\mathrm{UD}}^{C}\right), \end{array}$$

**Figure 4:**
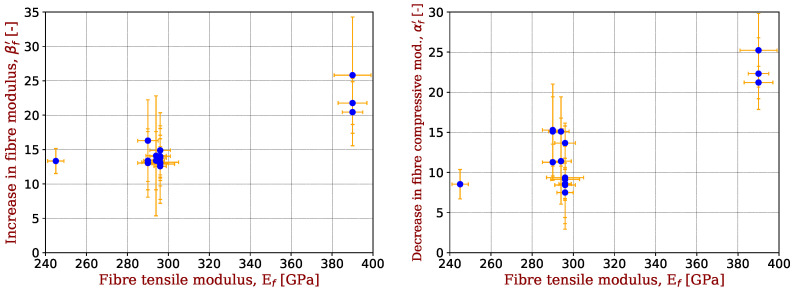
Non-linear elastic parameters of carbon fibres in compression ($\beta_{f}^{\prime }$) (**left**) and ($\alpha_{f}^{\prime }$) in compression (**right**).

In doing so, the coefficients $\alpha_{f}^{\prime }=\frac{\alpha_{\mathrm{UD}}}{E_{\mathrm{UD}}^{C0}}$ and $\beta_{f}^{\prime }=\frac{\beta_{\mathrm{UD}}}{E_{\mathrm{UD}}^{T0}}$ are introduced. They allow for $\beta_{f}^{\prime }$ values to be compared with values obtained in tensile tests on individual fibres [19] or on tows [9], which has been performed favourably by Keryvin et al. [13]. This possibility should not be taken for granted for $\alpha_{f}^{\prime }$, because the experimental difficulties of carrying out compression tests on µm-sized fibres are innumerable [11,12,17]. These parameters also get rid of the fibre volume fraction of UD plies. They are added to Table 2 and represented in Figure 4 as a function of the carbon fibre tensile modulus E${}_{f}$ known from the suppliers’ datasheets.

SM (one fibre) and IM fibres have a $\beta_{f}^{\prime }$ value of ∼13. HM (1 fibre) fibres have a $\beta_{f}^{\prime }$ value of ∼23. There is some scatter for IM fibres but $\alpha_{f}^{\prime }$ values between 7 and 15 are found, while they are 8 and 23 for the SM and HM fibres, respectively. A clear difference between SM/IM and HM fibres is evidenced.

### 4.2. Microstructure of Carbon Fibres

PAN-based carbon fibres have a unique microstructure that consists of carbon crystallite layers, crystallite disorder regions (amorphous phase) and needle-like microvoids [29]. The basic structural unit of carbon fibres is the graphene sheet. These sheets are preferably arranged parallel to the axis of the carbon fibre. Stacks of graphene sheets form crystallites, which are arranged either randomly or symmetrically with respect to the fibre axis. The most important difference between crystallites and graphite is that two adjacent sheets have no correlation of orientation or position. This is known as the turbostratic structure [30]. Correlations can be made between the mechanical properties with the microstructural features of carbon fibres, such as the degree of crystallinity or the size of crystallites. Loidl et al. [31] showed, for PAN-based fibres, that the increase in processing temperature from low for SM fibres (around 1800 °C) to high for HM fibres (2400 °C) passing through intermediate for IM fibres (2100 °C) results in a decrease in the interlayer spacing, and an increase in crystallite size and in crystallinity. The tensile modulus increases with the increase in three factors of the crystallites: their aspect ratio, their volume fraction and their degree of orientation [32]. A skin–core description of the fibres, combining amorphous and crystalline regions in the fibre core with a skin layer exhibiting a higher degree of orientation, was proposed by Kobayashi et al. [33] and can help in understanding recent results of mechanical properties of the core of fibres [12,34].

The high degree of crystallite orientation in HM fibres could be responsible for higher values of $\beta_{f}^{\prime }$ and $\alpha_{f}^{\prime }$. Following Ozcan et al. [35], SM and IM fibres similar to ours have misorientation angles (angle between crystallite orientation and fibre direction) of ∼16°, while HM fibres like HR40 fibre have a much lower angle of ∼10°. Increasing the already very oriented HR40 fibre will dramatically increase the stiffness since the crystallites are extremely anisotropic, while the increase would be more moderate for SM/IM fibres. This would explain the values of $\beta_{f}^{\prime }$, as now explained in more detail.

The elastic of a carbon fibre $E_{f}$ has been proposed [30] to depend on the tensile ($e_{\mathrm{cr}}$) and shear ($e_{\mathrm{cr}}$) moduli of the crystallites and of their orientation vis-à-vis the fibre axis, as shown in Equation (4):

(4) $$E_{f}\;\left(\theta \right)\;=\frac{1}{e_{\mathrm{cr}}+g_{\mathrm{cr}}\left(Z_{2}\;\left(\theta \right)\;-Z_{4}\;\left(\theta \right)\;\right)},$$

where *Z*${}_{2}$ and *Z*${}_{4}$ are the second- and fourth-order cosine moments of the crystallite orientation distribution θ with respect to the fibre axis. As a first approximation, they will be taken for a constant θ orientation distribution so that *Z*${}_{2}=\cos^{2}\theta$ and *Z*${}_{4}=\cos^{4}\theta$. The initial orientation of crystallites for carbon fibres similar to ours has been reported by Ozcan et al. [35]. The moduli of the crystallites are taken from Paris and Peterlik [30] with $e_{\mathrm{cr}}$ = 700 GPa and $g_{\mathrm{cr}}$ = 24 GPa. To obtain the changes in mean orientation angle in tension, we use the following method:

(i) Selection of the different fibres UTS50 (SM), IM2C (IM) and HR40 (HM);
(ii) Determination of their initial mean orientation angle via Equation (4) and their initial tensile modulus $E_{f}$ from Table 1;
(iii) Computation of the tensile modulus at 0.5% strain for each fibre via $\beta_{f}^{\prime }$ from Table 1;
(iv) Determination of the mean orientation angle via Equation (4) and these tensile moduli at 0.5% strain;
(v) Computation of the change in mean orientation angle.

**Figure 5:**
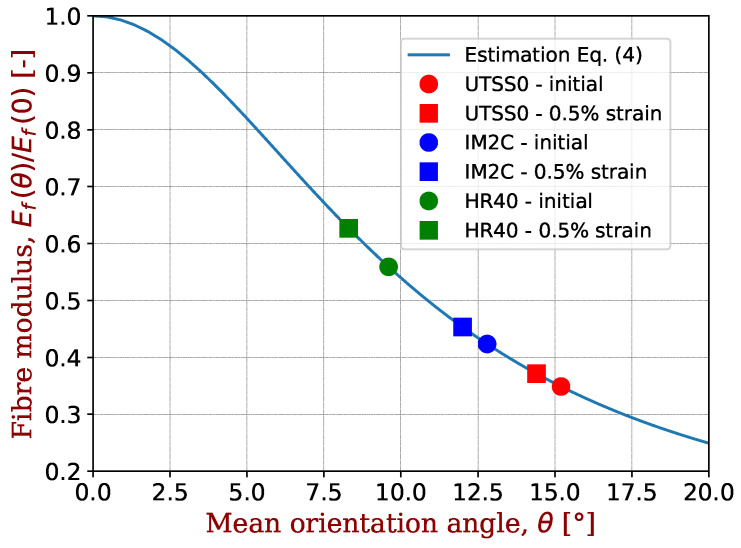
Changes in the orientation of crystallites at 0.5% strain in tension.

The results are presented in Table 3 and Figure 5.

The values of $\beta_{f}^{\prime }$ for tensile strains of 0.5% correspond to a change in the mean orientation angle of crystallites of −0.8°, −0.8° and −1.3° for UTS50, IM2C and HR40 fibres, respectively. This quantifies and validates our preceding comment.

As for compression, the higher oriented the crystallites, the higher their propensity to buckle, and therefore to obtain a softer behaviour [36]. This would explain higher values of $\alpha_{f}^{\prime }$ for HR40 fibre as compared to SM/IM fibres. A quantitative analysis such as that made for $\beta_{f}^{\prime }$ above for $\alpha_{f}^{\prime }$ remains to be conducted.

### 4.3. Signature (or Trace) of a Carbon Fibre during Bending Tests

**Figure 6:**
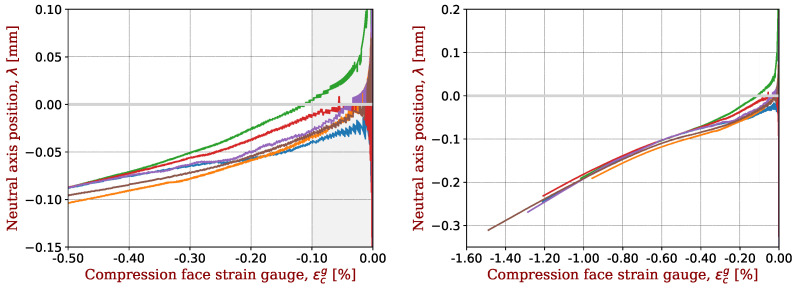
Shift in the neutral axis for CFRP with IM2C fibres in the range of determination of the non-linear elastic parameters (**left**) or in the full test range (**right**). A representative sample was taken for each CFRP and plotted with a given colour.

The shift in the neutral axis of bending samples with respect to the neutral axis is shown in Figure 6 for all composites with IM2C fibres. For the seven composites, this shift is extremely reproducible, as shown in Figure 6 (right). A magnification on the range of compressive strains above −0.5% is shown in Figure 6 (left). At this scale, some slight differences may be seen, especially for low values of strain. This comes from the fact that for low values of strains, the denominator of λ (see Equation (1)) is very small, and small errors have large consequences. This is why we selected strains below −0.1% for extracting parameters. Moreover, a very slight asymmetry for the plates, or even a different polishing when gluing the strain gauges on the two sides of samples, may result in a initial shift of the neutral axis, as reported by Marchandise et al. [24].

**Figure 7:**
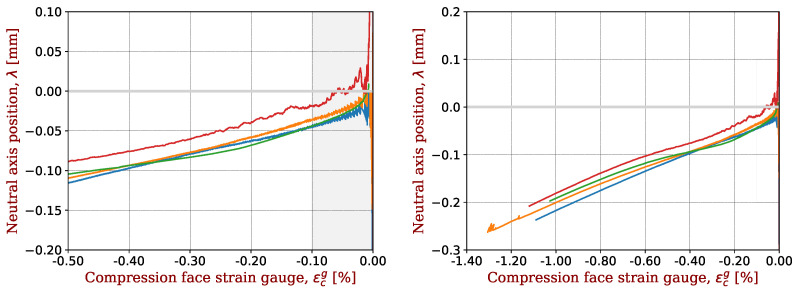
Shift in the neutral axis for CFRP with IMS65 fibres in the range of determination of the non-linear elastic parameters (**left**) or in the full test range (**right**). A representative sample was taken for each CFRP and plotted with a given colour.

**Figure 8:**
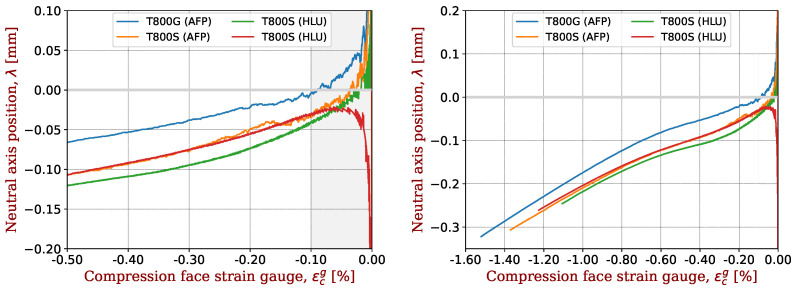
Shift in the neutral axis for CFRP with T800 fibres in the range of determination of the non-linear elastic parameters (**left**) or in the full test range (**right**). A representative sample was taken for each CFRP and plotted with a given colour.

**Figure 9:**
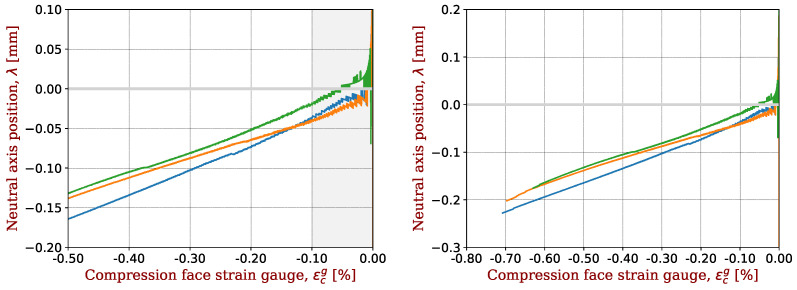
Shift in the neutral axis for CFRP with HR40 fibres in the range of determination of the non-linear elastic parameters (**left**) or in the full test range (**right**). A representative sample was taken for each CFRP.

The same plots are made for IMS65 (Figure 7), T800 (Figure 8) and HR40 (Figure 9). For T800 fibres, there is a noticeable difference between T800S (3 fibres) and T800G (1 fibre); see Figure 8.

**Figure 10:**
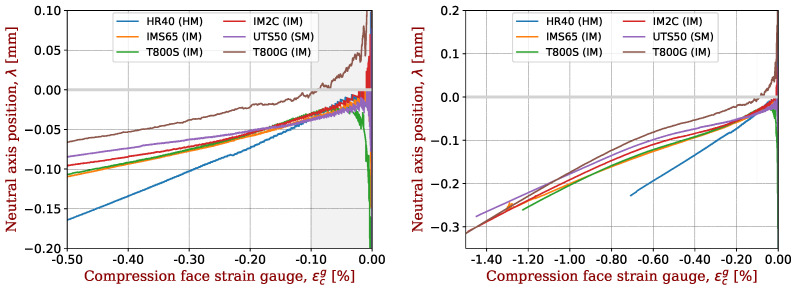
Shift in the neutral axis for CFRP studied in the range of determination of the non-linear elastic parameters (**left**) or in the full test range (**right**). A representative sample was taken for each carbon fibre.

All these plots give a high degree of confidence in the reproducibility of our approach, since the displacement of the neutral axis is a clear indicator of the elastic non-linearity of the UD and the fibres. A comparison between the different fibres is made in Figure 10. The offset is smaller for SM fibres than for IM fibres and, in turn, smaller than for HM fibres. The SM and IM fibres show a non-linear trend in this offset over the whole strain range. The slope is steeper at higher strains. In contrast, HM fibres show a linear shift, fully justifying the assumptions of Equation (2). This allows us to associate a sort of signature (or trace) with each fibre in terms of the behaviour of the deviation from the neutral axis. Following the analysis in Section 4.1 and Figure 5, it could possible to analyse the non-linear evolution of this offset over the full range of strains for the SM and IM fibres.

### 4.4. Insights from This Work

The scope of this work is threefold. Firstly, an experimental database is constructed, introducing the axial non-linear elastic properties: on the one hand, for composites with continuous carbon fibres and a polymer matrix, and on the other hand, for the carbon fibres themselves. Constructed using a single four-point bending test on a large number of different carbon fibres, this database is one of the original features of this paper.

Secondly, a discussion is conducted on the intrinsic signature of a carbon fibre constituted by the neutral fibre offset during flexural tests on composite laminates. Tensile stiffening is linked to the reorientation of graphene sheets, and the decrease in the angle with respect to the fibre axis is quantified.

Finally, from an engineering application point of view, there are several aspects for taking account this elastic non-linearity, which has been quantified and can be used for many composites thanks to this work, in the design of composite structures. From a deformation (or stiffness) design point of view, it is obvious that fine designs requiring precise control of deformations need to take this non-linearity into account [15]. In the case of stability design (or structural deployment via the triggering of instabilities, e.g., on thin structures) [22], this knowledge is indispensable. Finally, for strength-based designs, this non-linearity must be taken into account both in the measurement of strength and in the calculation of structures [14]. The level of non-linearity will depend on the loading mode on a laminate. Localising stresses in plies, one is concerned only in tensile and compressive longitudinal stresses.

## 5. Conclusions

A database of non-linear elastic parameters in tension and compression was provided for continuous carbon fibre polymer composites and carbon fibres of different stiffnesses. To our knowledge, such a database on non-linear parameters of a range of different carbon fibres does not exist. Four-point bending tests were used to demonstrate the non-linear elastic behaviour of composites. Assuming a linear evolution with strain of elastic moduli in tension and compression, and therefore a quadratic evolution of stresses with strains, the parameters expressing this non-linear elastic behaviour were identified. For comparing composites with carbon fibres of different failure strains, a specific range of identification was chosen below 0.5%. The composites manufactured were selected to be symmetrical in order to avoid disturbances, especially for low strains. This method enabled us to identify the parameters both for tension and compression in a single test.

The displacement (or shift) of the neutral axis from the mid-axis of the bending samples was considered to be an indicator of the non-linear elastic behaviour of CFRP. The trend of this displacement as a function of applied load was found to be the signature (or trace or imprint) of a given carbon fibre with its unique characteristics related to its ultra-microstructure. It was shown that the identified non-linear elastic parameters of UD depend only on those of the carbon fibres themselves. The non-linear elastic parameter in compression is important, because it is particularly difficult to determine using conventional fibre tests. An increase in tensile stiffness and a decrease in compressive stiffness have been correlated with a decrease in the average orientation angle of graphene sheets within crystallites. HM fibres show a much higher degree of non-linearity, linked to the decrease in the angle of orientation, which is already low, compared with SM or IM fibres.

The signature of each carbon fibre shows that for some of them, the displacement of the neutral axis is non-linear for the entire range of deformations up to failure. It might be interesting to study this non-linear elastic behaviour for strains greater than 0.5%. In this case, the possible contributions of fibre waviness, negligible for low strains, could then play a more important role.

## Figures and Tables

**Table 1:** Results of data analysis from bending tests on different CFRP including parameters $E_{\mathrm{UD}}^{T0}$, $E_{\mathrm{UD}}^{C0}$, $\beta_{\mathrm{UD}}$ and $\alpha_{\mathrm{UD}}$ (see main text for details). E${}_{f}$ is the fibre tensile modulus known from suppliers’ datasheets. The manufacturing is also indicated by HLU (hand lay-up) or AFP.

Fibre	E${}_{f}$	Matrix	Manuf.	$E_{\mathrm{UD}}^{T0}$	$E_{\mathrm{UD}}^{C0}$	$\beta_{\mathrm{UD}}$	$\alpha_{\mathrm{UD}}$
Name	[GPa]	Name	Process	[GPa]	[GPa]	[GPa/%]	[GPa/%]
UTS50	245	RV101	AFP	165 ± 4	164 ± 3	22 ± 3	14 ± 3
IMS65	290	DT120	HLU	142 ± 2	139 ± 4	19 ± 6	21 ± 6
IMS65	290	BT080	HLU	135 ± 5	133 ± 3	22 ± 8	15 ± 3
IMS65	290	DT124	HLU	161 ± 5	157 ± 4	21 ± 8	24 ± 9
T800S	294	MR074	AFP	152 ± 3	151 ± 3	14 ± 2	23 ± 1
T800S	294	DT120	HLU	142 ± 4	139 ± 4	19 ± 6	21 ± 6
T800S	294	DT124	HLU	149 ± 5	149 ± 7	21 ± 13	17 ± 8
T800G	294	MR074	AFP	143 ± 4	143 ± 4	20 ± 3	8 ± 3
IM2C	296	Se84LV	AFP	162 ± 4	160 ± 3	21 ± 4	12 ± 5
IM2C	296	Se84nano2	HLU	167 ± 3	163 ± 3	23 ± 11	14 ± 3
IM2C	296	Se84nano2	HLU	175 ± 9	171 ± 9	23 ± 6	16 ± 11
IM2C	296	M81	AFP	168 ± 5	166 ± 5	25 ± 6	14 ± 8
IM2C	296	M79	HLU	164 ± 5	161 ± 5	23 ± 5	22 ± 4
IM2C	296	R374-1	HLU	155 ± 7	153 ± 3	20 ± 8	14 ± 4
HR40	390	Se84nano2	HLU	213 ± 9	218 ± 6	55 ± 18	55 ± 10
HR40	390	Se84nano2	HLU	225 ± 5	224 ± 5	46 ± 11	50 ± 10
HR40	390	R374-1	HLU	193 ± 7	198 ± 4	42 ± 6	42 ± 4

**Table 2:** Non-dimensionless non-linear elastic parameters of carbon fibres $\beta_{f}^{\prime }=\frac{\beta_{\mathrm{UD}}}{E_{\mathrm{UD}}^{T0}}$ and $\alpha_{f}^{\prime }=\frac{\alpha_{\mathrm{UD}}}{E_{\mathrm{UD}}^{C0}}$ (see Equation (3)). E${}_{f}$ is the fibre tensile modulus known from suppliers’ datasheets.

Fibre	E${}_{f}$	$\beta_{f}^{\prime }$	$\alpha_{f}^{\prime }$
Name	[GPa]	[−]	[−]
UTS50	245	13 ± 2	9 ± 2
IMS65	290	13 ± 4	15 ± 4
T800G	294	14 ± 4	6 ± 4
T800S	294	12 ± 4	12 ± 4
IM2C	296	14 ± 2	8 ± 3
HR40	390	23 ± 8	23 ± 5

**Table 3:** Changes in the orientation of crystallites at 0.5% strain in tension. The fibres of the present study are associated to similar fibres from which the initial mean orientation of crystallites is known [35].

Fibre	$E_{f}$	Initial	$\beta_{f}^{\prime }$	Orientation	Change
Studied	Associated	Orientation		@0.5% Strain	in Angle
	[GPa]	[°]	[−]	[°]	[°]
UTS50	245	15.2	13	14.4	−0.8
IM2C	296	12.8	14	12.0	−0.8
HR40	390	9.6	23	8.3	−1.3

## Data Availability

Data will be made available on request.

## Abbreviations

The following abbreviations are used in this manuscript:

CFRP	Carbon fibre-reinforced plastics
UD	Unidirectional lamina
SM	Standard-modulus carbon fibre
IM	Intermediate-modulus carbon fibre
HM	High-modulus carbon fibre
PAN	Polyacrylonitrile precursor
AFP	Automated fibre placement
HLU	Hand lay-up

## References

[1] Njuguna J. (2016). Lightweight Composite Structures in Transport.

[2] Argon A., Herman H. (1972). Fracture of Composites. Treatise on Materials Science and Technology.

[3] Wisnom M.R. (1992). On the high compressive strains achieved in bending test on unidirectional carbon-fibre/epoxy. Compos. Sci. Technol..

[4] Vandreumel W.H.M., Kamp J.L.M. (1977). Non-Hookean Behavior in Fiber Direction of Carbon-Fiber Composites and Influence of Fiber Waviness on Tensile Properties. J. Compos. Mater..

[5] Wisnom M.R. Limitations of Linear Elastic Bending Theory Applied to Four Point Bending of Unidirectional Carbon Fibre-Epoxy. Proceedings of the 31st Structures, Structural Dynamics and Materials Conference.

[6] Csanádi T., Németh D., Zhang C., Dusza J. (2017). Nanoindentation derived elastic constants of carbon fibres and their nanostructural based predictions. Carbon.

[7] Guruprasad T., Keryvin V., Charleux L., Guin J.P., Arnould O. (2021). On the determination of the elastic constants of carbon fibres by nanoindentation tests. Carbon.

[8] Curtis G.J., Milne M.J., Reynolds W.N. (1968). Non-Hookean Behaviour of Strong Carbon Fibres. Nature.

[9] Hughes J. (1986). Strength and modulus of current carbon fibres. Carbon.

[10] Shioya M., Hayakawa E., Takaku A. (1996). Non-hookean stress-strain response and changes in crystallite orientation of carbon fibres. J. Mater. Sci..

[11] Ueda M., Akiyama M. (2019). Compression test of a single carbon fiber in a scanning electron microscope and its evaluation via finite element analysis. Adv. Compos. Mater..

[12] Guruprasad T., Keryvin V., Kermouche G., Marthouret Y., Sao-Joao S. (2023). Compressive behaviour of carbon fibres micropillars by in situ SEM nanocompression. Compos. Part A Appl. Sci. Manuf..

[13] Keryvin V., Marchandise A., Grandidier J.C. (2022). Non-linear elastic longitudinal behaviour of continuous carbon fibres/epoxy matrix composite laminae: Material or geometrical feature?. Compos. Part B Eng..

[14] Keryvin V., Marchandise A., Mechin P.Y., Grandidier J.C. (2020). Determination of the longitudinal non linear elastic behaviour and compressive strength of a {CFRP} ply by bending tests on laminates. Compos. Part B Eng..

[15] Keryvin V., Méchin P.Y. Mast design performance—Influence of the non-linear elastic behaviour of carbon fibre composite plies on the accuracy of fluid/structure interaction models for estimating mast bending. Proceedings of the High Performance Yacht Design Conference HPYD8.

[16] Nakatani M., Shioya M., Yamashita J. (1999). Axial compressive fracture of carbon fibers. Carbon.

[17] Sugimoto Y., Kato T., Shioya M., Kobayashi T., Sumiya K., Fujie M. (2013). Structure change of carbon fibers during axial compression. Carbon.

[18] Nunna S., Ravindran A.R., Mroszczok J., Creighton C., Varley R.J. (2023). A review of the structural factors which control compression in carbon fibres and their composites. Compos. Struct..

[19] Kant M., Penumadu D. (2014). Dynamic mechanical characterization for nonlinear behavior of single carbon fibers. Compos. Part A Appl. Sci. Manuf..

[20] Allix O., Ladevèze P., Vittecoq E. (1994). Modelling and identification of the mechanical behaviour of composite laminates in compression. Compos. Sci. Technol..

[21] Montagnier O. (2005). Compression Characterization of High-modulus Carbon Fibers. J. Compos. Mater..

[22] Murphey T.W., Peterson M.E., Grigoriev M.M. (2015). Large Strain Four-Point Bending of Thin Unidirectional Composites. J. Spacecr. Rockets.

[23] Devaux H., Balze R., Robert S. (2012). Finite-element model of a rigid wing sail for a maxi trimaran. Mech. Ind..

[24] Marchandise A., Keryvin V., Grohens Y., Borgne R.L. (2023). Influence of the lay-up and curing steps in the manufacturing process of thick laminate composites on their compressive strength. Compos. Part A Appl. Sci. Manuf..

[25] Mechin P., Keryvin V., Grandidier J., Glehen D. (2019). An experimental protocol to measure the parameters affecting the compressive strength of CFRP with a fibre micro-buckling failure criterion. Compos. Struct..

[26] Djordjević I.M., Sekulić D.R., Stevanović M.M. (2007). Non-linear elastic behaviour of carbon fibres of different structural and mechanical characteristic. J. Serbian Chem. Soc..

[27] Mujika F., Carbajal N., Arrese A., Mondragon I. (2006). Determination of tensile and compressive moduli by flexural tests. Polym. Test..

[28] Serna Moreno M.C., Romero Gutiérrez A., Martínez Vicente J.L. (2016). Different response under tension and compression of unidirectional carbon fibre laminates in a three-point bending test. Compos. Struct..

[29] Park S.J. (2018). Carbon Fibres.

[30] Paris O., Peterlik H., Paris O. (2016). Single Carbon Fibres: Structure from X-ray Diffraction and Nanomechanical Properties. Structure and Multiscale Mechanics of Carbon Nanomaterials.

[31] Loidl D., Paris O., Burghammer M., Riekel C., Peterlik H. (2005). Direct Observation of Nanocrystallite Buckling in Carbon Fibers under Bending Load. Phys. Rev. Lett..

[32] Zhong Y., Bian W. (2017). Analysis of the tensile moduli affected by microstructures among seven types of carbon fibers. Compos. Part B Eng..

[33] Kobayashi T., Sumiya K., Fukuba Y., Fujie M., Takahagi T., Tashiro K. (2011). Structural heterogeneity and stress distribution in carbon fiber monofilament as revealed by synchrotron micro-beam X-ray scattering and micro-Raman spectral measurements. Carbon.

[34] Chen L., Hao L., Liu S., Ding G., Sun X., Zhang W., Li F., Jiao W., Yang F., Xu Z. (2020). Modulus distribution in polyacrylonitrile-based carbon fiber monofilaments. Carbon.

[35] Ozcan S., Vautard F., Naskar A.K. (2014). Designing the Structure of Carbon Fibers for Optimal Mechanical Properties. PolymER Precursor-Derived Carbon.

[36] Tanaka F., Okabe T., Okuda H., Ise M., Kinloch I.A., Mori T., Young R.J. (2013). The effect of nanostructure upon the deformation micromechanics of carbon fibres. Carbon.

